# Weaning assessment of veno-arteriovenous (V-AV) extracorporeal membrane oxygenation (ECMO)

**DOI:** 10.1186/s40635-023-00538-y

**Published:** 2023-08-01

**Authors:** Johannes Heymer, Anna Hein, Matthias Ott, Tobias Schilling, Daniel Räpple

**Affiliations:** Klinikum Stuttgart, Stuttgart, Germany

**Keywords:** V-AV ECMO, Weaning, Back flow, Off-sweep

## Abstract

Currently, there is a lack of methods for simultaneous assessment of readiness for decannulation of the veno-venous (V-V) and veno-arterial (V-A) components during veno-arteriovenous (V-AV) extracorporal membrane oxygenation (ECMO) support. We describe a novel approach using a simultaneous off-sweep and controlled backflow test to assess readiness for decannulation from V-AV ECMO. This method needs testing in future clinical trials.

V-AV ECMO is a configuration for extracorporeal life support (ECLS) in combined heart and lung failure [1]. Although numerous methods have been described to assess readiness for decannulation from veno-arterial (V-A) ECMO and veno-venous (V-V) ECMO, there is a lack of methods for simultaneous assessment of the V-V and V-A components during V-AV ECMO support.

Readiness for decannulation from V-A ECMO can be evaluated through a controlled backflow trial, which creates an artificial A-V fistula through the ECMO circuit [2, 3]. Decannulation readiness from V-V ECMO can be assessed via an off-sweep trial, thereby eliminating respiratory support. V-A support is regarded as a contraindication for an off-sweep trial because it would result in the so-called inverse Harlequin phenomenon by providing only deoxygenated blood to the lower body circulation through the arterial cannula.

We propose that readiness for decannulation from V-AV ECMO can be assessed through a simultaneous A-V backflow and off-sweep test. We describe how this test can be performed using the Maquet Cardiohelp device (Getinge, Rastatt, Germany). To conduct the test, ECMO flow should be reduced to a minimal total flow of approximately 2.5 L/min to prevent clotting in low-flow conditions. The settings for rotations per minute will be maintained at that constant value for the entire procedure. After reducing ECMO flow, the flow sensor is applied to the arterial line. Then, the adjustable clamp of the venous return line is opened until the flow in the arterial line reverses. By opening the adjustable clamp, the flow in the venous return line increases and the pressure in the venous return line decreases. Once the pressure falls below the mean arterial pressure, the flow in the arterial line reverses. To avoid the ECMO device switching to backflow prevention mode, the flow sensor should now be rotated 180 degrees to align with the outflow direction from the artery. The venous return line of the V-V component is further opened to allow a reverse flow in the arterial line of approximately 5 mL/kg/min. The setup now corresponds to a regular backflow test (Fig. 1). The backflow test assesses if the patient tolerates the removal of circulatory support and provides an additional load to the right ventricle. Since there is no flow from the ECMO to the arterial circulation at this time, the sweep can be turned off to enable a simultaneous off-sweep trial. In this configuration there is neither respiratory nor circulatory support, so a global weaning assessment is possible.

**Fig. 1:**
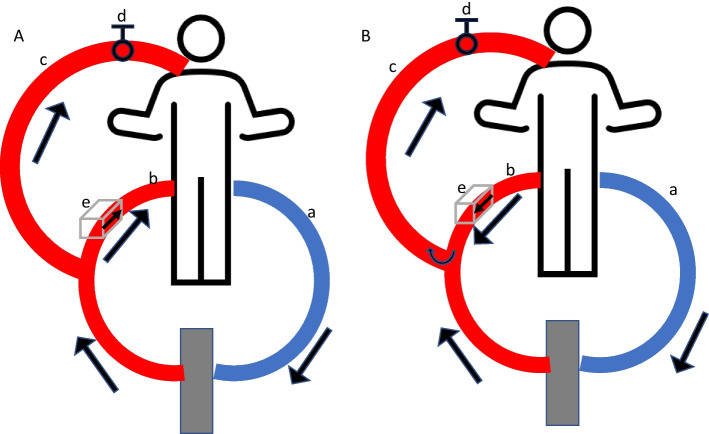
Schematic representation of flow. **A** Regular V-AV configuration, **B** direction of flow during controlled back flow. a: venous cannula in IVC via femoral vein; b: arterial cannula via femoral artery; c: arterial cannula in internal jugular vein; d: adjustable clamp; e: flow sensor with direction

We believe this test is appropriate for a highly selected subgroup of patients with initially severe pathology (e.g., patients undergoing extracorporeal cardiopulmonary resuscitation (ECPR) with concomitant acute pulmonary pathology) that has been fully reversed. In this subgroup, our proposed algorithm allows for a rapid assessment of readiness for decannulation and, therefore, prompt and complete removal of the device, thus minimizing the inherent risks of ECMO. If the timing of improvement of respiratory and circulatory failure differs, the simultaneous weaning assessment as described above may multiply the risk of weaning failure.

To test our hypothesis, that the above described configuration allows for a more rapid assessment of readiness for decannulation, this approach could be tested in a clinical trial against a previously described stepwise approach [1] after the safety of our configuration has been assessed in observational trials or case series. At the moment, there is no scientific evidence to support the weaning strategy we described.

## Data Availability

Not applicable.

## Abbreviations

ECMO: Extracorporal membrane oxygenation
V-V: Veno-venous
V-A: Veno-arterial
V-AV: Veno-arteriovenous
ECLS: Extracorporal life support
ECPR: Extracorporal cardiopulmonary resuscitation
